# Factors associated with clearance of hepatitis B virus surface antigen in patients infected with human immunodeficiency virus

**DOI:** 10.1097/MD.0000000000021271

**Published:** 2020-07-17

**Authors:** Takeya Tsutsumi, Hidenori Sato, Tadashi Kikuchi, Kazuhiko Ikeuchi, Lay Ahyoung Lim, Eisuke Adachi, Michiko Koga, Kazuya Okushin, Takuya Kawahara, Tomohiko Koibuchi, Hiroshi Yotsuyanagi

**Affiliations:** a Division of Infectious Diseases, Advanced Clinical Research Center, Institute of Medical Science, The University of Tokyo; b Department of Infectious Diseases and Applied Immunology, IMSUT Hospital of The Institute of Medical Science, The University of Tokyo; c Department of Infection Control and Prevention, Graduate School of Medicine, The University of Tokyo; d Central Coordinating Unit, Clinical Research Support Center, The University of Tokyo Hospital, Tokyo, Japan.

**Keywords:** HBsAg clearance, HBV, HIV, host factors

## Abstract

Owing to similar routes of transmission, hepatitis B virus (HBV) and human immunodeficiency virus (HIV) coinfection commonly occurs. Compared with patients infected with only HBV, coinfected patients develop persistent HBV infection followed by advanced liver diseases. However, the characteristics of HIV-infected patients who can achieve the clearance of HBV surface antigen (HBsAg) have not been clarified. In this study, we retrospectively examined patients coinfected with HBV and HIV and determined the host factors associated with HBsAg clearance.

Among HIV-infected patients who visited our hospital between 1994 and 2017, we examined medical records of those who were seropositive for HBsAg at least once. Among them, patients who cleared HBsAg afterward were regarded as “cured,” while those who remained HBsAg-seropositive until 2017 were “chronic.”

HBsAg seropositivity was found in 57 patients, and among them, 27 male patients were cured whereas 18 were chronic. The cured patients were significantly younger and had higher CD4 cell and platelet counts than the chronic patients. In addition, the cured patients had higher levels of transaminases after the detection of HBsAg. Multivariate analysis revealed age as an independent factor. Analyses of the patients infected with genotype A also showed that the cured patients had significantly higher CD4 cell counts.

Considering that the CD4 cell and platelet counts were higher in the cured patients, immunological and liver functions were closely associated with HBsAg clearance. Higher levels of transaminases in the cured patients may also reflect the immunological function leading to HBsAg clearance.

## 1 Introduction

Since the hepatitis B virus (HBV) and human immunodeficiency virus (HIV) are transmitted from human to human by similar pathways, patients with HIV infection are at a high risk of HBV coinfection^[1,2]^ as well as other sexually transmitted infections.^[3]^ According to the data from Western countries, the rate of coinfection varies among risk categories. The rate is highest among men who have sex with men (MSM), slightly lower among intravenous drug users, and much lower in individuals infected through heterosexual contacts.^[4–7]^ Generally, HIV-infected patients more rapidly progress from chronic HBV infection to liver cirrhosis, end-stage liver diseases, and hepatocellular carcinoma than only-HBV-infected patients.^[8]^ In addition, a recent study showed that the seropositivity for the antibody against the HBV core antigen (HBcAb) is correlated with poor HIV viremia control,^[9]^ suggesting that the HBV coinfection may also influence the HIV control and subsequent HIV-induced pathogenesis.

In non-HIV-infected individuals, acute hepatitis caused by HBV infection of the non-A genotype in adulthood is usually transient and most of the patients can clear HBV surface antigen (HBsAg) within 6 to 12 months, which is called “functional cure.” Ito et al^[10]^ reported that 9 out of 312 patients diagnosed as having acute hepatitis B remained HBsAg-seropositive after 12 months of acute infection and that 8 out of the 9 persistently HBsAg-positive patients were infected with HBV of genotype A. The rates of chronicity were 0.1% (1 among 105 patients) for the non-A genotype and 7.5% (8 among 107 patients) for the genotype A. In the presence of HIV infection, the rate of spontaneous clearance of HBsAg is lower than in the absence of HIV infection.^[11]^ In addition, HIV-infected patients have more opportunities for acute HBV infection owing to their unique lifestyle. Therefore, vaccination of non-HBV-immunized HIV-infected patients is recommended to prevent acute HBV infection, although the efficacy of HBV vaccines is low in such patients.^[12,13]^ Considering these situations, it is important to determine the characteristics of HIV-infected patients who would develop chronic HBV infection in view of the preventive strategies including antiretroviral therapy (ART), which is also effective for HBV.

Therefore, in this study, we retrospectively examined the clinical data of HIV-infected patients who were previously HBsAg-seropositive during their visits to our hospital. Some patients cleared HBsAg afterward, while others remained HBsAg-seropositive. By comparing these patients, we determined the host factors associated with HBsAg clearance. The findings will be useful for identifying HIV-infected patients who are potentially prone to develop chronic HBV infection and accordingly for educating individual patients at high risk in a clinical setting.

## 2 Methods

### 2.1 Patients

Between 1994 and 2017, 979 HIV-infected patients visited the affiliate hospital of The Institute of Medical Science, The University of Tokyo (IMSUT Hospital). We retrospectively examined the laboratory data of these patients, and patients lacking HBV-related tests such as tests for HBsAg, the antibody against HBsAg (HBsAb), and HBcAb were excluded from the analysis. We also excluded patients with hemophilia because of the possibility that they may be infected with HIV and HBV through tainted blood products; therefore, the period of HBV infection may be longer in hemophilic patients than in nonhemophilic patients. Concerning patients who were seropositive for HBsAg at least once in their clinical courses, their baseline and subsequent clinical data were collected. Patients who had already started ART before their first visit to IMSUT Hospital were excluded since their baseline immunological and virological data were modified by the ART. Among patients who met the above criteria, those whose HBsAg disappeared afterward were regarded as “cured,” while those who remained seropositive for HBsAg until 2017, for at least 6 months, were regarded as having “chronic” HBV infection. Patients who stopped visiting IMSUT Hospital before confirming the HBsAg seroconversion were also excluded. The study was approved by the Ethics Committee of the Institute of Medical Science, The University of Tokyo (Study Permission No. 30-109-B20190402).

### 2.2 Laboratory tests

Most of the laboratory tests were performed at IMSUT Hospital. Titers of HBsAg, HBsAb, and HBcAb were measured by enzyme immunoassay, counting immunoassay, or chemiluminescent assay, depending on the period of the examination. The plasma HIV-RNA load and HBV genotype were examined by an outside company. The detection limits for the HIV-RNA load were 400 copies/mL in the period between 1994 and March 2001, 50 between April 2001 and December 2007, 40 between January 2008 and September 2011, and 20 from October 2011 to 2017.

### 2.3 Statistical analysis

Data processing and analyses were performed using JMP Pro 14 software (SAS Institute Japan, Tokyo, Japan). In univariate analyses, the following continuous variables were compared using Wilcoxon rank-sum test for the HBs antigen status: age, CD4 cell count, CD8 cell count, HIV-RNA load, platelet count, and peak levels of aspartate aminotransferase (AST) and alanine aminotransferase (ALT). In multivariate analysis, logistic regression analysis was performed to identify significant and independent associations between each variable and the HBs antigen status. A *P* value < .05 was considered to indicate statistical significance. All data are shown as the median and the interquartile range unless otherwise stated.

## 3 Results

**Figure 1:**
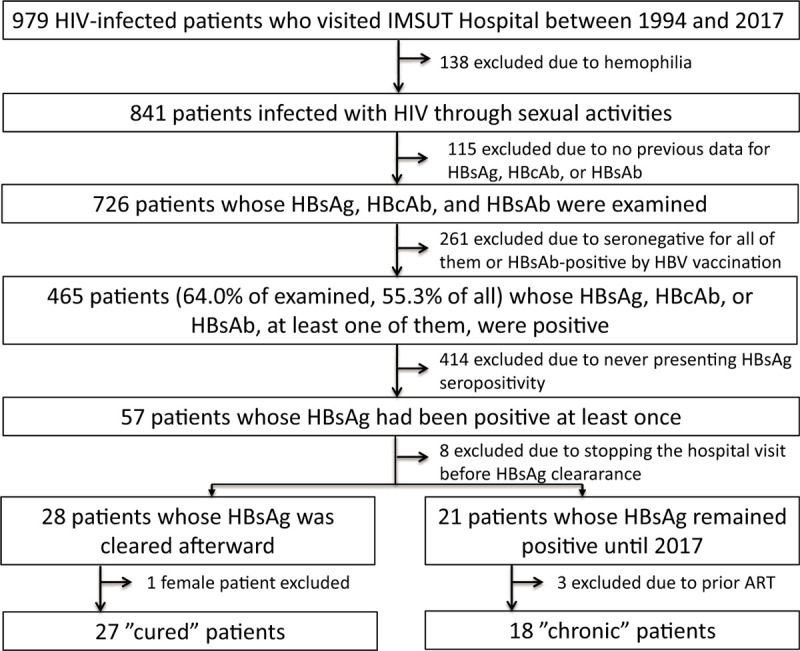
Protocol for selecting patients coinfected with HIV and HBV. HBV = hepatitis B virus, HIV = human immunodeficiency virus.

From 1994 to 2017, a total of 979 HIV-infected patients had visited our hospital. There were 138 patients with hemophilia, and the remaining 841 patients were probably infected with HIV through sexual activities; 1 patient also declared intravenous drug use. Among them, 726 patients were examined for HBsAg, HBsAb, and HBcAb (Fig. 1). Among the patients, 465 (64.0% of those examined, 55.3% of all patients) were seropositive for at least HBsAg, HBcAb, or HBsAb; we excluded patients who were positive only for HBsAb presumably due to vaccination. Most of the patients were positive only for HBcAb, and some were positive for both HBsAb and HBcAb. HBsAg seropositivity was observed in 57 patients at least once in their clinical courses. Among the patients who had been seropositive for HBsAg, 28 showed the disappearance of HBsAg, while 21 showed the presence of HBsAg until 2017. The remaining 8 patients stopped their visit to our hospital before the confirmation of the HBsAg disappearance. All of the patients except 1 cured patient were male, and 3 chronic patients were already receiving ART before coming to our hospital. Therefore, we proceeded with the following analysis with 27 cured male patients and 18 chronic male patients.

Clinical data of the patients at their first visit to our hospital are shown in Table 1. The cured patients were significantly younger than the chronic patients. The CD4 cell and platelet counts were higher in the cured patients than in the chronic patients. On the other hand, the CD8 cell count, the CD4/CD8 ratio, and the HIV viral load were not significantly different between the 2 groups. The number of patients with a history of AIDS was not also significantly different. Table 2 shows the clinical data of these patients at the onset of and after HBsAg was detected. Since the presence of HBsAg was identified simultaneously with HIV infection in 14 cured and 11 chronic patients, the values of factors such as the cell and platelet counts and the HIV-RNA load were very similar to those at their first visit. At the timepoint of HBsAg seropositivity, the platelet and the CD4 cell counts were significantly higher in the cured patients; moreover, the cured patients were younger than the chronic patients, as similarly observed at their first visit. The CD8 cell count, CD4/CD8 ratio, and HIV-RNA load were also not significantly different between the 2 groups. After the HBsAg detection, the peak levels of AST and ALT in the cured patients were significantly higher than those in the chronic patients. Concerning the HBV genotype, the ratio of patients with genotype A was higher in the cured group (18 among 19 patients) than in the chronic group (10 among 16 patients) as far as we compared the number of patients whose HBV genotype was determined. All of the cured patients started ART after the clearance of HBsAg; therefore, it is not possible that the ART contributed to at least the HBsAg clearance in the cured patients.

**Table 1:**
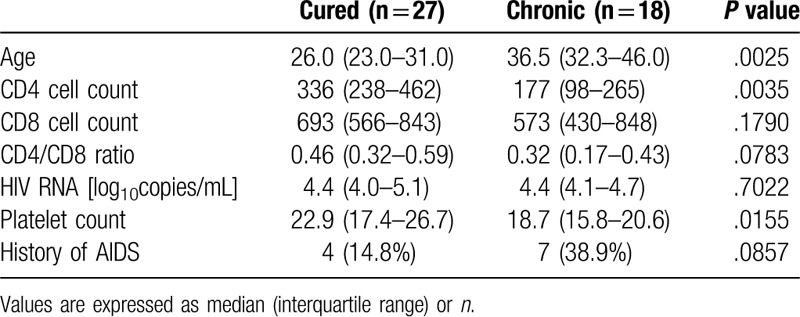
Background characteristic of the patients at 1st visit.

**Table 2:**
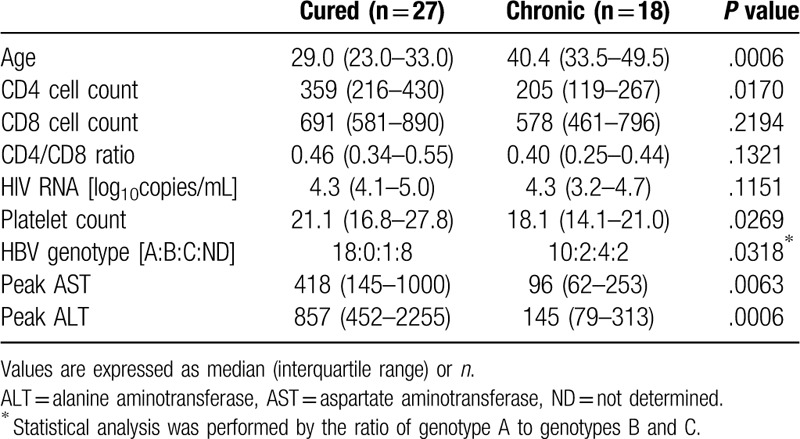
Clinical data of the patients at the onset of and after the detection of HBsAg seropositivity.

There was no information about the exact route of HBV infection in most of these patients. As shown in Table 2, many patients were infected with HBV of genotype A but others were infected with HBV of genotype B or C. It is speculated that most of the patients were infected with HBV by sexual activities or other routes such as drug injection in adulthood, and actually, we could confirm the HBsAg-seropositivity of their mother in none of these patients as far as we carefully investigated the clinical record. However, we could not exclude the possibility that some patients were infected with HBV in childhood by vertical or horizontal transmission, at least in the case of genotype B or C because these genotypes are predominant among patients with maternal-infant transmission in Asia including Japan.^[14]^ In the case of vertical transmission or horizontal transmission in childhood, it is uncommon that HBsAg disappears naturally even in patients without HIV infection. Since the findings that HBV of genotype B or C was more prevalent in the chronic patients suggest the possibility that there might be some patients who were infected in childhood in the chronic patients, we similarly examined patients infected with HBV of genotype A since these patients might have been infected with HBV by sexual transmission or other humoral routes in adulthood. There were 18 cured and 10 chronic patients infected with genotype A, and the clinical data at the onset of HBsAg detection and subsequent peak levels of AST and ALT were compared. As shown in Table 3, only the CD4 cell count was significantly higher in the cured patients than in the chronic patients. The cured patients were younger and showed higher ALT levels than the chronic patients, but the differences did not reach statistical significance, probably owing to the small number of patients. On the other hand, the platelet counts, which were higher in the cured patients when compared regardless of the HBV genotype, were similar between the groups.

**Table 3:**
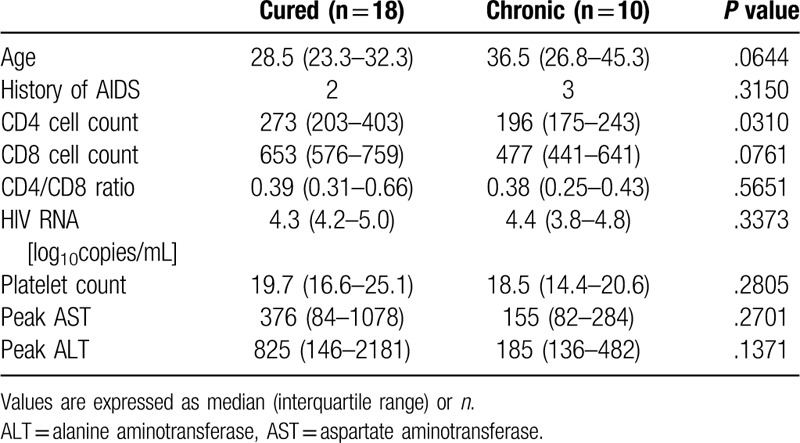
Clinical data of the patients infected with HBV of genotype A.

Finally, we tried to identify the independent factors associated with the clearance of HBsAg. Multivariate analysis of factors in all patients revealed that only age was an independent factor (*P* = .0300), that is, younger patients generally achieved the clearance of HBsAg. However, when the analysis was limited to patients with HBV of genotype A, no independent factors were identified.

## 4 Discussion

In this study, we retrospectively examined HIV-infected patients and found that about 64.0% of them had been infected with HBV, which was lower than the rate reported from Italy showing that 81.2% of HIV-positive inmates had current or past HBV infection.^[15]^ Most of these patients were seropositive only for HBcAb and seronegative for HBsAg, indicating that these patients were previously infected with HBV and had cleared HBsAg, indicating their “functional cure.” The number of patients who presented HBsAg-seropositive at least once in their clinical courses was 57, 7.9% among patients examined for HBV-related laboratory tests. This rate was a little bit higher than that reported by Phinius et al^[16]^ showing 27 (6.2%) among 435 HIV-infected patients in Botswana experienced HBsAg-seropositive during 4-year period, possibly due to the difference in the observation period. Among the 57 male patients who were previously HBsAg-seropositive, 27 became HBsAg-seronegative afterward and 21 remained HBsAg-seropositive until recently. The remaining 9 patients stopped visiting the hospital before the confirmation of seroconversion. By comparing the 27 cured and 18 chronic patients who were ART-naive when HBsAg was detected, we found that the cured patients were significantly younger and had higher CD4 cell and platelet counts. In addition, the cured patients had significantly higher peak levels of ALT and AST after the detection of HBsAg. The higher CD4 and platelet counts may reflect the well-preserved host immune status and the degree of liver fibrosis, respectively. Accordingly, the levels of hepatic transaminases may be higher in patients with immunologically good condition. Concerning this point, we have recently reported that in HIV-infected patients with acute hepatitis C, the peak ALT levels positively correlated with the CD4/CD8 ratio, which is a biomarker of immunosenescence in HIV-infected adults.^[17]^ These findings suggest that HBsAg clearance is associated with the immunological and liver conditions of HIV-infected patients. From another point of view, when we see an HIV-infected patient with HBV coinfection, we may be able to estimate the possibility of HBV chronicity by his or her age and CD4 cell count and make a therapeutic strategy against HBV as well as HIV, since some anti-HIV drugs are also useful for HBV treatment. In this point, a recently reported 2-drug regimen that is safe and cost-effective should be refrained due to the low genetic barrier against HBV.^[18]^

When only the patients infected with HBV of genotype A were compared, the cured patients also showed a significantly higher CD4 cell count, and tended to be younger and to show higher peak levels of ALT than the chronic patients, similarly to the comparison of all patients. However, the platelet count was not significantly different between the cured and chronic patients. On the basis of these findings, we considered that the grade of liver fibrosis might not be associated with HBsAg clearance, at least in the case of HBV infection of genotype A. Otherwise, there is a possibility that some chronic patients infected with genotype B or C might be infected with HBV at birth or in childhood and had chronic hepatitis for a relatively long period, which led to the decrease in their platelet count. However, the platelet count was almost similar between the chronic patients with genotype A (mean, 17.0; median, 18.5) and those with genotype B or C (mean, 16.0; median, 14.8), suggesting that this possibility is low.

There are several studies of patients coinfected with HBV and HIV. Jaroszewicz et al^[19]^ examined the HBsAg levels in HIV and HBV coinfected patients in Taiwan and found that HBsAg level negatively correlated with CD4 cell count. In addition, the HBsAg levels were significantly lower in patients with a greater increase in CD4 cell count after the ART; however, they found that the drug regimen did not affect the HBsAg levels. On the other hand, Gatanaga et al^[20]^ examined 33 HIV-infected patients with HBV incident infections who were naive or received ART without anti-HBV drugs. Among these patients, 13 developed chronic HBV infection, while the other 20 patients had a transient HBV infection. The median CD4 cell count was lower in the patients with chronic infection than in those with transient infection, although the difference was not significant (*P* = .068). Also, in our present study, the CD4 cell count was higher in the cured patients than in the chronic patients. These findings also indicate that HIV-related immunological abnormalities may play a role in the induction of chronic HBV infection.

Multivariate analysis revealed that only age was an independent factor for the clearance of HBsAg. In HIV-infected patients, older age is a strong predictor of accelerated progression of HIV disease even in the presence of ART.^[21]^ In addition, HIV-infected patients are prone to developing liver diseases from not only common causes such as alcohol abuse, NAFLD, viral hepatitis, and aging, but also HIV-specific causes such as systemic chronic inflammation, HIV-induced direct liver injury, and ART-related toxicity.^[22]^ Furthermore, Kim et al^[23]^ reported that poorly controlled HIV monoinfection itself is an independent risk factor for liver fibrosis. Considering these situations, liver fibrosis may be associated with HIV-related immunological conditions, and finally, age may mainly contribute to the immune and liver functions.

Among non-HIV-infected patients with acute hepatitis B in adulthood in Japan, most of them experienced transient infection and cleared HBsAg within 6 months. But recently, many studies have shown that the infection with HBV of genotype A sometimes led to chronic infection.^[10,24–26]^ Ito et al examined the risk factors for the long-term persistence of HBsAg after acute HBV infection in Japanese adults and demonstrated that the rate of progression to chronicity, defined as the persistence of HBsAg positivity for more than 6 or 12 months, was higher in patients infected with genotype A. They also showed by multivariate logistic regression analysis that only genotype A was independently associated with viral persistence after the acute infection. Furthermore, ALT levels were significantly higher in patients who cleared HBsAg within 6 or 12 months after the acute infection than in those who could not clear HBsAg in the same period.^[10]^ Also in our present study, the peak levels of ALT and AST were significantly higher in the cured patients with HIV infection. These results suggest that stronger inflammation induced by host immune response may contribute to the down-regulation of HBV, leading to the elimination of HBsAg.

Concerning the host genomic factor, several studies demonstrated by a genome-wide association study that single-nucleotide polymorphism (SNPs) in human leukocyte antigen (HLA)-DPA1 (rs3077) and HLA-DPB1 (rs9277535) loci are associated with HBV clearance in Asian people without HIV infection.^[27,28]^ Therefore, we determined these SNPs in 19 cured and 11 chronic patients whose serum stocks were available with informed consent. However, there was no significant difference in the frequency of SNPs in either of these alleles between the cured and chronic patients as far as we investigated (Tsutsumi, 2019, unpublished data). Although the number of patients examined was rather small, the SNPs in HLA-DPA1 and HLA-DPB1 might not be associated with HBsAg clearance in HIV-infected patients since these patients may have defective immune functions even in the presence of ART.

Our study has several limitations. First, in this study, only a small number of patients in a single center were analyzed. In addition, patients visiting our hospital between 1994 and 2017 were recruited in this study, and the therapeutic strategies against HIV have greatly changed over this period, which may affect HIV-related immune and liver functions. Second, the onset of HBV infection was unclear and some patients might be infected with HBV at birth through vertical transmission or in childhood through horizontal transmission. In such patients, HBV infection tends to be chronic and the clearance of HBsAg in adulthood is rare even without HIV infection. Indeed, the number of patients with genotypes B and C was significantly larger in the chronic patients than in the cured patients, and some of these patients might be infected at birth or in childhood before HIV infection. Third, HBV-related virological and serological markers such as HBeAg, HBeAb, HBcAb, and HBV viral load were not considered owing to the lack of previous medical records. Finally, this study is a retrospective study based on the database; therefore, it is possible that some important information that may influence the outcome of this study may be lacking. These include the information about the adherence to HIV treatment, the comorbid diseases and drugs, and the accurately timed laboratory examinations, especially HBV-related tests.

In this study, we examined the rate of HBV coinfection among HIV-infected patients and found that about 64.0% of them had been infected with HBV. This rate is very high compared with non-HIV-infected people; therefore, we should take into account the possibility of the potential HBV coinfection when we start to treat an HIV-infected patient. We also compared the clinical data between HIV-infected patients who were persistently HBsAg-positive and those who cleared HBsAg. The patients who cleared HBsAg were significantly younger, and showed higher CD4 cell and platelet counts and peak levels of ALT and AST, suggesting that these patients had well-preserved immune and hepatic functions compared with the HIV-infected patients with chronic HBV infection. Owing to the lifestyle of HIV-infected MSM, the opportunities to become infected with HBV, especially HBV of genotype A, are not rare; therefore, it is important to maintain a good immune function to prevent chronic HBV infection.

## Acknowledgments

The authors thank Ms Tomoe Senkoji for her helpful assistance and Myu Research (http://www.myu-inc.jp/myuresearch.html) for the review of English.

## Author contributions

**Conceptualization:** Takeya Tsutsumi, Hiroshi Yotsuyanagi.

**Data curation:** Hidenori Sato, Tadashi Kikuchi, Kazuhiko Ikeuchi, Lay Ahyoung Lim, Eisuke Adachi, Michiko Koga, Tomohiko Koibuchi.

**Formal analysis:** Takeya Tsutsumi, Kazuya Okushin, Takuya Kawahara.

**Investigation:** Takeya Tsutsumi, Hidenori Sato, Tadashi Kikuchi, Kazuhiko Ikeuchi, Lay Ahyoung Lim, Eisuke Adachi, Michiko Koga, Tomohiko Koibuchi.

**Methodology:** Takeya Tsutsumi, Kazuya Okushin, Takuya Kawahara.

**Project administration:** Hiroshi Yotsuyanagi.

**Writing-original draft:** Takeya Tsutsumi.

**Writing-review & editing:** Hiroshi Yotsuyanagi.
